# Infundibular origin of a duplicated anterior communicating artery mimicking an aneurysm

**DOI:** 10.1007/s00276-025-03703-y

**Published:** 2025-08-30

**Authors:** George Triantafyllou, Nikolaos-Achilleas Arkoudis, Ornella Moschovaki-Zeiger, Christos Koutserimpas, Georgios Velonakis, Maria Piagkou

**Affiliations:** 1 Department of Anatomy, Faculty of Health Sciences, School of Medicine, National and Kapodistrian University of Athens, 75 Mikras Asias Str., Goudi, 11527 Athens, Greece; 2 Research Unit of Radiology and Medical Imaging, National and Kapodistrian University of Athens, Athens, Greece; 3 2nd Department of Radiology, General University Hospital “Attikon”, National and Kapodistrian University of Athens, Athens, Greece; 4 School of Rehabilitation Health Sciences, University of Patras, Patras, Greece

**Keywords:** Anterior communicating artery, Cerebral arterial circle, Variation, Artery duplication, Infundibular dilation, Magnetic resonance angiography

## Abstract

The anterior communicating artery (AComA) is a common site of morphological variation and aneurysm formation. We report an unusual vascular anomaly identified during routine 3T magnetic resonance angiography (MRA) in a 67-year-old male under evaluation for cerebrovascular disease. Imaging revealed a duplicated AComA, with the first segment originating from an infundibular dilation of the right anterior cerebral artery (ACA). A second, extremely short AComA segment, 0.9 mm, was located distally. Although infundibular dilations are typically benign, they warrant careful evaluation due to their potential to mimic aneurysms and possibly contribute to aneurysm development. Accurate identification using high-resolution imaging techniques is essential to prevent misdiagnosis and to inform appropriate clinical management. This case highlights the importance of recognizing rare vascular variants, particularly in preoperative planning and differential diagnosis.

**Supplementary Information:**

The online version contains supplementary material available at 10.1007/s00276-025-03703-y.

## Introduction

An increasing number of imaging scans are performed on the cerebral arterial circle to investigate cerebrovascular diseases clinically. These processes effectively demonstrate the typical anatomy and variants of these vessels.

The anterior circulation comprises the anterior cerebral artery (ACA) and the middle cerebral artery (MCA), both emanating from the internal carotid artery (ICA). The bilateral ACAs are interconnected through the anterior communicating artery (AComA) [7]. The anterior circulation exhibits significant morphological variability, as numerous variants have been documented for each vessel [7]. A recent meta-analysis of our research team indicated that the AComA morphological variations have a pooled prevalence of approximately 33%. Nonetheless, it remains the most prevalent site for intracranial aneurysms [8].

Infundibular dilatations are funnel-shaped enlargements that should be considered normal anatomical variants and not be confused with pathological conditions, such as aneurysms [5]. Herein, we report the coexistence of infundibular dilation and variation of the AComA.

## Anatomic variation

During routine examination for possible cerebrovascular disease, the scan of a 67-year-old male patient was further investigated. A magnetic resonance angiography (MRA) was examined at three Tesla (3T), while the imaging protocol was performed using the time-of-flight technique (TOF) with a Philips 3T Achieva TX MRI scanner (Philips, Best, the Netherlands) with an 8-channel head coil. The scans were documented using Horos software version 3.3.6 (Horos Project). Evidence was gathered from the multiplanar reconstruction of the axial, coronal, and sagittal slices and their three-dimensional volume reconstruction.

**Fig. 1:**
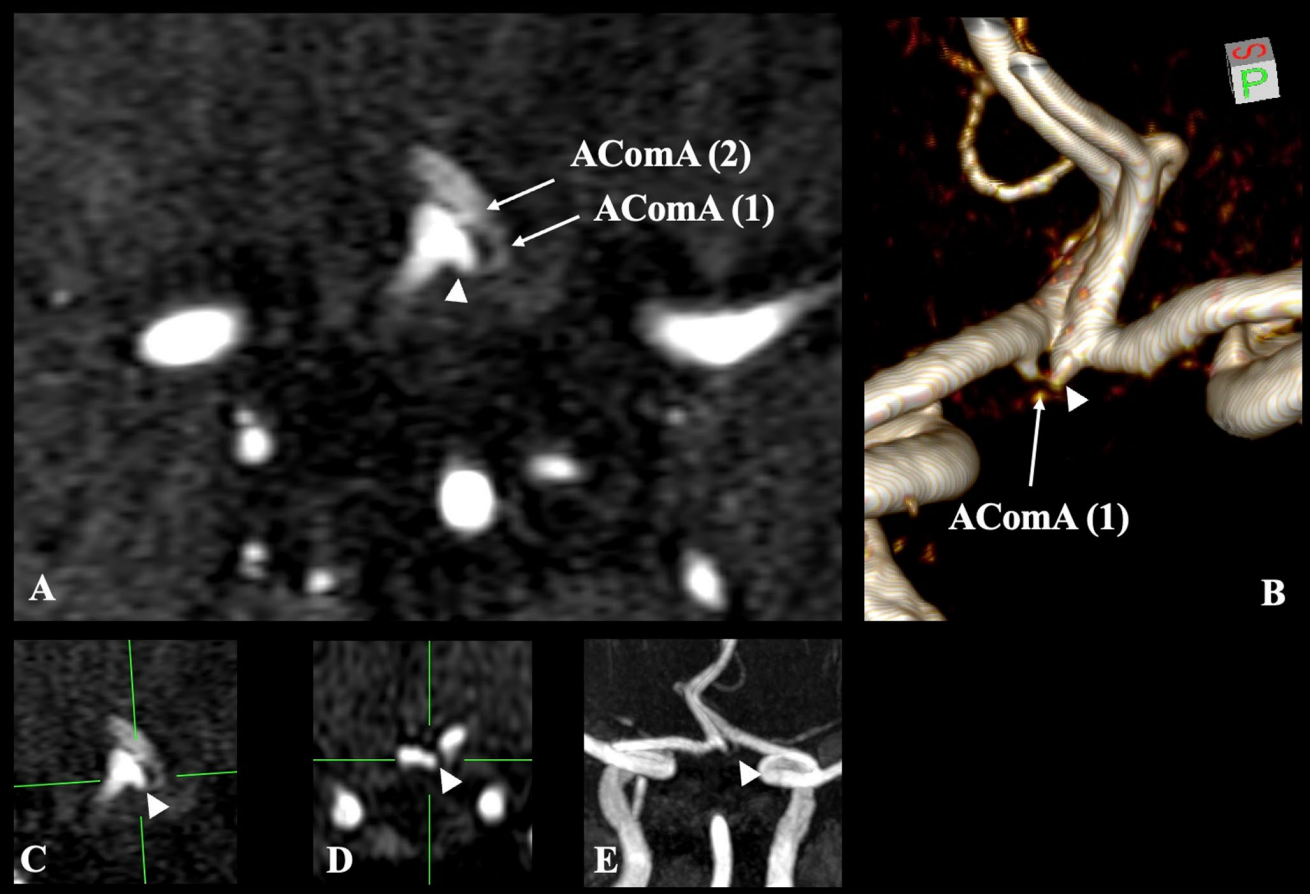
Imaging of a Duplicated Anterior Communicating Artery (AComA) with Infundibular Origin. **A** Axial slice displays the duplicated AComA: AComA (1) arises from an infundibular dilation (white arrowhead), while AComA (2) appears distally. **B** Three-dimensional volume rendering shows the origin of AComA (1) from the infundibular dilation (arrowhead), highlighting the funnel-shaped configuration. **C**, **D** Axial and coronal reconstructions further illustrate the infundibular dilation (arrowheads) and the origin of AComA (1), although the small vessel caliber limits visualization clarity. **E** Axial slice with maximum intensity projection depicting the duplicated AComA with Infundibular Origin

Regarding the anterior circulation, the ACA typically originates from the ICA bilaterally. Distally to its origin (17.2 mm of length), the right ACA had an infundibular dilation (1.31 mm diameter), giving rise to an AComA of 2.0 mm. The infundibular origin had a funnel shape, while the AComA was originated from the dilatation apex. Distally to this vessel (2.84 mm of length), another extremely short AComA (0.9 mm of length) was present (Fig. 1A–E, Supplementary Material). These images confirm the presence of a duplicated AComA with one vessel having infundibular origin, which mimicked a saccular aneurysm (Fig. 1A–E, Supplementary Material). The variation was depicted in axial slices and three-dimensional reconstructions; however, due to the small caliber of the vessels described, it was not effectively demonstrated in sagittal and coronal. The posterior circulation and the remainder of the cerebral arterial circle exhibited no variants.

## Discussion

The present imaging report indicates the existence of a duplicate AComA with an infundibular origin, while one of the branches was significantly shorter. During embryological development, the AComA initially manifests in a plexiform configuration at an embryonic length of 18 mm. The artery attains its final morphology in embryos measuring between 21 and 24 mm (approximately 44 days of gestation). Nevertheless, any deviation from this developmental process may result in variations, such as the duplicate form observed in the current case [1].

Numerous morphological variations of the AComA are documented in the existing literature. Triantafyllou et al. [8] classified nine types of variants in their meta-analysis. Typical morphology is associated with a pooled prevalence of 67.32%; thus, variants are observed in 32.68%, indicating a common location for anatomical variations. The prevalence of duplicate AComA is reported to be 4.3%, with a significant difference noted between the designs of studies (cadaveric versus imaging) and nationalities [8]. Furthermore, the mean length was determined to be 2.84 mm [8], suggesting that the second AComA in the current case can be considered exceedingly short. Another variation resembling AComA duplication- via imaging techniques- is the fenestration of the vessel, with a pooled prevalence of 5% [8]. Uchino et al. [9] demonstrated that AComA fenestration can only be identified through the enhanced imaging quality of 3 T MRA. They also illustrated variants that may be mistakenly diagnosed for fenestration, including duplication, partial duplication, and fenestration at the A1-A2 junction [9]. Additionally, Endo et al. [2] recently identified the coexistence of AComA duplication with accessory MRA wherein the duplicate AComA mimicked an aneurysm, similar to the case currently under discussion.

The AComA duplication may have limited clinical implications by itself, while duplication and fenestration were linked with aneurysm formation [8]. However, this case’s clinical significance is associated with the presence of the infundibulum origin and the differential diagnosis for aneurysms. Concerning the presence of infundibula, there are few reports documenting their occurrence in conjunction with arterial variants. Narducci et al. [6] detailed an infundibular origin of an accessory anterior cerebral artery during routine evaluation of the MRA of a 19-year-old female patient. Kihira et al. [4] identified an infundibulum located at the origin of an accessory MCA, whereas Endo et al. [3] observed the same phenomenon at the origin of a duplicate MCA. In our case, we reported on the infundibular origin of a duplicate AComA; however, our review of current literature yielded no similar cases. It is recommended that infundibula be diagnosed, particularly utilizing the 3 T field MRA, during periods of optimal background saturation [5]. The most prevalent location for such anomalies is the posterior communicating artery, which originates from the ICA [5]. There are three primary criteria for the identification of infundibula: (1) a size of less than 3 mm, (2) a funnel shape without a neck, and (3) a vessel emerging from its apex [5]. These criteria are instrumental for precise documentation and, more importantly, for the accurate differentiation of infundibula from aneurysms, as misdiagnosis may result in unnecessary further diagnostic procedures, some of which may be interventional (e.g., digital subtraction angiography—DSA).

Table 1 summarizes helpful differential diagnosis tips distinguishing between infundibula and saccular aneurysms. Infundibula are predominantly considered typical variants; however, there have been instances where they have progressed into an aneurysm that ultimately ruptured [5]. Consequently, they may represent a risk factor for aneurysm formation [5]. Thin-section CTA or high-resolution TOF-MRA can typically confirm the funnel shape and branch origin without requiring invasive angiography. Nevertheless, DSA remains the gold standard if CTA or MRA do not demonstrate the branch arising from the apex.

**Table 1:** Key Differentiating Features Between Infundibulum and Saccular Aneurysm [5]

Feature	Infundibulum	Saccular Aneurysm
Size	Typically, < 3 mm in maximum diameter	Usually > 3 mm (but may be smaller)
Shape	Funnel- or cone-shaped outpouching; no definable neck; gradual tapering from parent artery	Spherical or lobulated with a discrete neck
Branch origin	A branch vessel arises from the apex of the dilation	No branch arises from the apex; an aneurysm is eccentrically located

In conclusion, we have identified a duplicate AComA, one of which exhibited an infundibular origin resembling an aneurysm, while the other was notably short. Neuroradiologists and neurosurgeons must recognize and document rare variations of the cerebral arterial circle. Effectively demonstrating the vessels prior to surgical interventions is crucial, necessitating the employment of appropriate imaging techniques. For instance, in particular cases, the utilization of high-quality imaging (MRA with 3T) might be imperative.

## Supplementary Information

Below is the link to the electronic supplementary material.

Supplementary material: The axial slices of the Duplicated Anterior Communicating Artery (AComA) with Infundibular Origin. (MP4 2876 kb)

## Data Availability

No datasets were generated or analysed during the current study.
